# Fishing for vaccines against *Vibrio cholerae* using *in silico* pan-proteomic reverse vaccinology approach

**DOI:** 10.7717/peerj.6223

**Published:** 2019-06-19

**Authors:** Muhammad I. Rashid, Sammia Rehman, Amjad Ali, Saadia Andleeb

**Affiliations:** Department of Industrial Biotechnology, Atta ur Rahman School of Applied Biosciences, National University of Science and Technology, Islamabad, Pakistan

**Keywords:** *Vibrio cholerae*, Reverse vaccinology, Cholera, Peptide vaccine, Epitope prediction

## Abstract

**Background:**

Cholera, an acute enteric infection, is a serious health challenge in both the underdeveloped and the developing world. It is caused by *Vibrio cholerae* after ingestion of fecal contaminated food or water. Cholera outbreaks have recently been observed in regions facing natural calamities (i.e., earthquake in Haiti 2010) or war (i.e., ongoing civil war in Yemen 2016) where healthcare and sanitary setups have been disrupted as a consequence. Whole-cell oral cholera vaccines (OCVs) have been in market but their regimen efficacy has been questioned. A reverse vaccinology (RV) approach has been applied as a successful anti-microbial measure for many infectious diseases.

**Methodology:**

With the aim of finding new protective antigens for vaccine development, the *V. cholerae* O1 (biovar eltr str. N16961) proteome was computationally screened in a sequential prioritization approach that focused on determining the antigenicity of potential vaccine candidates. Essential, accessible, virulent and immunogenic proteins were selected as potential candidates. The predicted epitopes were filtered for effective binding with MHC alleles and epitopes binding with greater MHC alleles were selected.

**Results:**

In this study, we report lipoprotein *NlpD*, outer membrane protein *OmpU*, accessory colonization factor *AcfA*, Porin, putative and outer membrane protein *OmpW* as potential candidates qualifying all the set criteria. These predicted epitopes can offer a potential for development of a reliable peptide or subunit vaccine for *V. cholerae*.

## Introduction

*Vibrio cholerae* is a prominent waterborne facultative pathogen which causes cholera disease which causes extreme dehydration and loss of electrolytes in patients (Pal, 2014). Strains of *V. cholera* O1 and O139 can be choleragenic. Further on, O1 serogroup is divided into classical and El TOR biotypes (Finkelstein, 1996). Cholera is a notifiable endemic disease in developing and underdeveloped countries  (Charles et al., 2017; Chowdhury et al., 2017; Qin et al., 2017; Noora et al., 2017). *V. cholerae* infections are a major factor with estimated annual global mortality around >100,000 (Ali et al., 2012; Reilly, 2015). The current seventh cholera pandemic is reported to be caused by El Tor biotype strains while some regional epidemics have also been observed to have *V. cholerae* El Ttor biotype strains as causative agents (Karaolis, Lan & Reeves, 1995; Reidl & Klose, 2002; Levine et al., 1995). The toxigenic strains are capable of causing explosive outbreaks and epidemics in regions with devastated or poor sanitary infrastructure as observed in Haiti in 2010  (Barzilay et al., 2013; Jackson et al., 2013; O’Connor et al., 2011). Climate change and other factors have been noted to gain increased significance in outbreaks (Chowdhury et al., 2017; Bertuzzo & Mari, 2017). Recent outbreaks of cholera are a result of poor sanitation, environmental pollution, natural and manmade disasters and unavailability of clean drinking water in affected areas (Nelson et al., 2015; Muhjazi et al., 2017; Kennedy, Harmer & McCoy, 2017; Hendriksen et al., 2011). The emergence and widescale spread of antibiotic resistance in the last six decades has been a huge challenge (Marti, Variatza & Balcazar, 2014; Klontz et al., 2014; Shakerian et al., 2017). Antimicrobial resistance has generally been a hindrance to the effective therapy of infectious diseases for as long as antibiotics have been used (Mazel, 2006). Despite the fact that during cholera treatment the antibiotics are limited as an adjunct to re-hydration, antibiotic usage has been observed to shorten the disease duration by 50% (Roux et al., 2015). *V. cholerae* can also serve as reservoir for resistance mechanisms for horizontal transmission, as it is capable enough to procure and spread the resistance determinants via all forms of genetic transfer strategies (Gupta et al., 2016; Martinez-Urtaza et al., 2008; Sedas, 2007; Barati et al., 2015). A potent cholera vaccine could be effective in natural disasters or other humanitarian situations as it can provide immunity when given preventively.

Concurrent strategies have been aimed at development of oral formulations capable of imparting mucosal immunity. Few anti-cholera oral formulations were tested in humans. An early study developed formulations comprising of cholera toxin B-subunit and inactivated bacterial cells was tested from 1985 to 1989 in Bangladesh (Fournier & Villeneuve, 1998). A recent clinical trial administered O-specific polysaccharide (OSP) to human subjects and demonstrated anti-OSP and vibriocidal antibody responses (Islam et al., 2018). In another study long-term efficacy and protection was assessed for killed bivalent, whole-cell oral cholera vaccine in Haiti  (Franke et al., 2018). This study reported a decrease in the effectiveness of single dose oral vaccines in comparison to two doses over the period of 4 years. Currently, various strategies have been employed to develop live attenuated cholera vaccines. A recently published study reported development of a genetically engineered *V. cholerae* O1 strain CVD 103-HgR as a live attenuated vaccine (Kaper et al., 1994). A recently published Phase 3 clinical trial (NCT02094586) of live oral cholera vaccine reported a 94% vibriocidal antibody seroconversion rate 6 months post-vaccination (McCarty et al., 2018). This single dose cholera vaccine was developed using attenuated recombinant *Vibrio cholerae* O1 vaccine strain CVD 103-HgR. This clinical trial recruited over 3,000 adult volunteers with 90% more efficacy in comparison to placebo group. One serious concern is regarding the safety of the vaccines, and similar formulations had faced efficacy and performance issues (Charles et al., 2017; Richie et al., 2000; Koelle et al., 2005). The possibility of horizontal gene transfer and reversion of live attenuated vaccine forms back to wild types with virulence spectrum and antibiotic resistance could aggravate the situation (Frey, 2007). Under special conditions, viral live attenuated vaccines have been reported to result in adverse effects (Moro et al., 2011; Lauring, Jones & Andino, 2010; Keller-Stanislawski et al., 2014).

A reverse vaccinology (RV) approach is the vaccine development strategy in the genomics era. This approach predicts vaccine candidates by screening genome and proteome, evaluates using algorithms and computational tools for proteins with best suitable properties as a potential vaccine agent (Rappuoli, 2000). In contrast to conventional vaccine development strategies, RV strategy provides rapid vaccine design and reduces the dependency on conventional animal testing based screening for getting a potentially suitable candidate. A number of vaccines have been developed for pathogens such as *Streptococcus pneumoniae*  (Wizemann et al., 2001), serogroup B *Neisseria meningitides* (MenB) (Pizza et al., 2000), *Cryptosporidium hominis* (Manque et al., 2011) , *Mycobacterium tuberculosis* (Betts, 2002), and *Bacillus anthracis* (Ariel et al., 2002).

This study is aimed at identification of extracellular and outer membrane proteins that can serve as better antigen targets for *V. cholerae*. We report lipoprotein NlpD, outer membrane protein OmpU, accessory colonization factor AcfA, Porin, putative and outer membrane protein OmpW as potential candidates qualifying all the set criteria. These predicted epitopes can offer a potential for development of a reliable peptide or subunit vaccine for *V. cholerae* in calamities hit regions as preemptive preventive protection. This is the first study to report vaccine target prediction using reverse vaccinology and reductive screening approach against *V. cholerae* O1 biovar El Tor.

## Methodology

**Figure 1:**
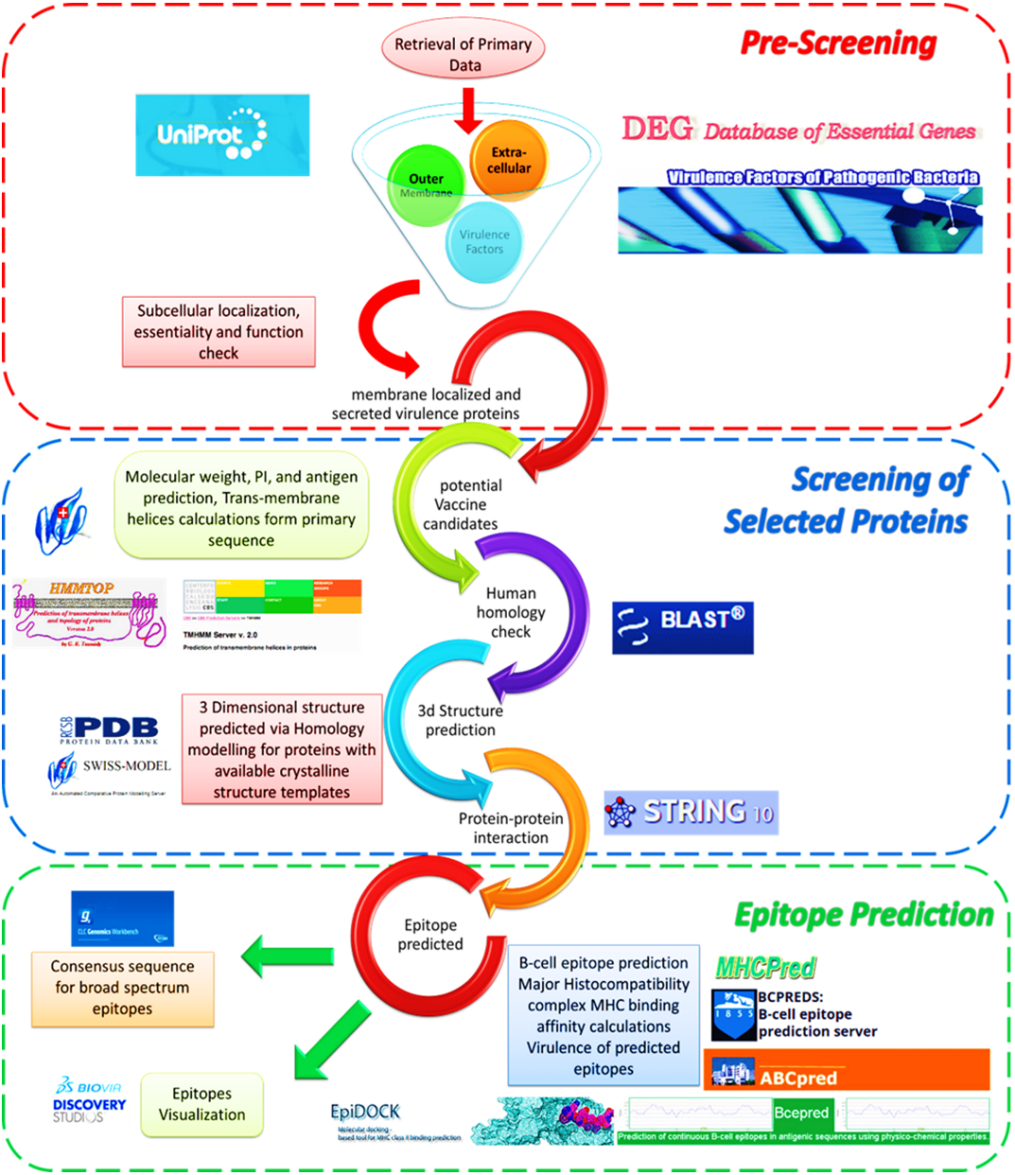
Reverse vaccinology screening process overview. Reverse vaccinology approach based computational framework for prediction of vaccine candidates for Vibrio cholerae O1 (biovar eltr str. N16961) (Richie et al., 2000).

We adopted our previously devised computational framework (Fig. 1) that includes three comprehensive steps for prediction of prospective vaccine candidates for *V. cholera* as described in detail (Rashid et al., 2017).

1. Pre-Screening of primary data
  The steps involved pre-screening of primary data include retrieval of the *V. cholerae* O1 (biovar eltr str. N16961) proteome from UniProt (Bairoch & Apweiler, 2000). Subcellular localization was predicted using the primary sequences of the *V. cholerae* proteome PSORTb V3.0 (Yu et al., 2010) and CELLO v2.5 (Yu, Lin & Hwang, 2004). Database of Essential Genes (DEG) (http://tubic.tju.edu.cn/deg/) version 10.4 provided the essentiality information of the proteins (Luo et al., 2014). The virulence check was performed using the virulence factor database (VFdb) for identification of potential virulence proteins (Chen et al., 2011). These steps were adopted to identify vital virulence proteins and respective epitopes to be subjected to peptide vaccine discovery.
2. Screening of selected proteins
  Screening of selected proteins was performed for their suitability of prospective immuno-protective potential. The criteria included appropriate molecular weight (<110 kDa estimated via ExPASy Compute pI / Mw Tool (Gasteiger et al., 2005)), prediction of antigenic and virulence potentials, protein structural details and human homologue search. The crystalline structures for these proteins were obtained from structural database Protein Data Bank (PDB) (Bernstein et al., 1977) or developed using the SWISS-MODEL server (Schwede et al., 2003) and interactions within the pathogen, and with host proteins and cluster of orthologous groups COG were studied using STRING (Search Tool for the Retrieval of Interacting Genes/Proteins) (Szklarczyk et al., 2011).
3. Epitope Selection
  In third step epitopes were predicted using multiple approaches via different algorithms in order to obtain broad spectrum epitopes. The predicted epitopes were screened to obtain epitopes capable of efficient binding to higher numbers of MHC alleles (Naz et al., 2015). *Continuous B-Cell Epitopes* were predicted using BcePred server  (Saha & Raghava, 2006). The BCPreds server was employed for prediction of 20-mer B-cell epitopes (EL-Manzalawy, Dobbs & Honavar, 2008). ABCpred, an artificial neural network based B-cell epitope prediction server, was also used for predicting B-cell epitopes  (Saha & Raghava, 2006). Default threshold values were used for each server. Propred and PropredI servers were used to investigate epitope interactions with MHC I and II alleles (Singh & Raghava, 2003), while antigenicity and IC_50_ calculations were performed with the help of MHCPred (Guan et al., 2003). Using proteins’ three dimensional epitopes were visualized using Discovery studio v4.1 (BIOVIA, 2015). Finally sequences of selected proteins from other virulent strains were obtained *from members of V. cholerae* NCBI Taxonomic group (TAXID: 666). The predicted antigenic regions were analyzed via BioEdit Sequence Alignment Editor, for sequence divergence against *V. cholerae* representative strains and consensus sequences were obtained for respective vaccine candidate for inter-strain immune-protection against *V. cholerae*.

## Results

### Primary data retrieval

**Figure 2:**
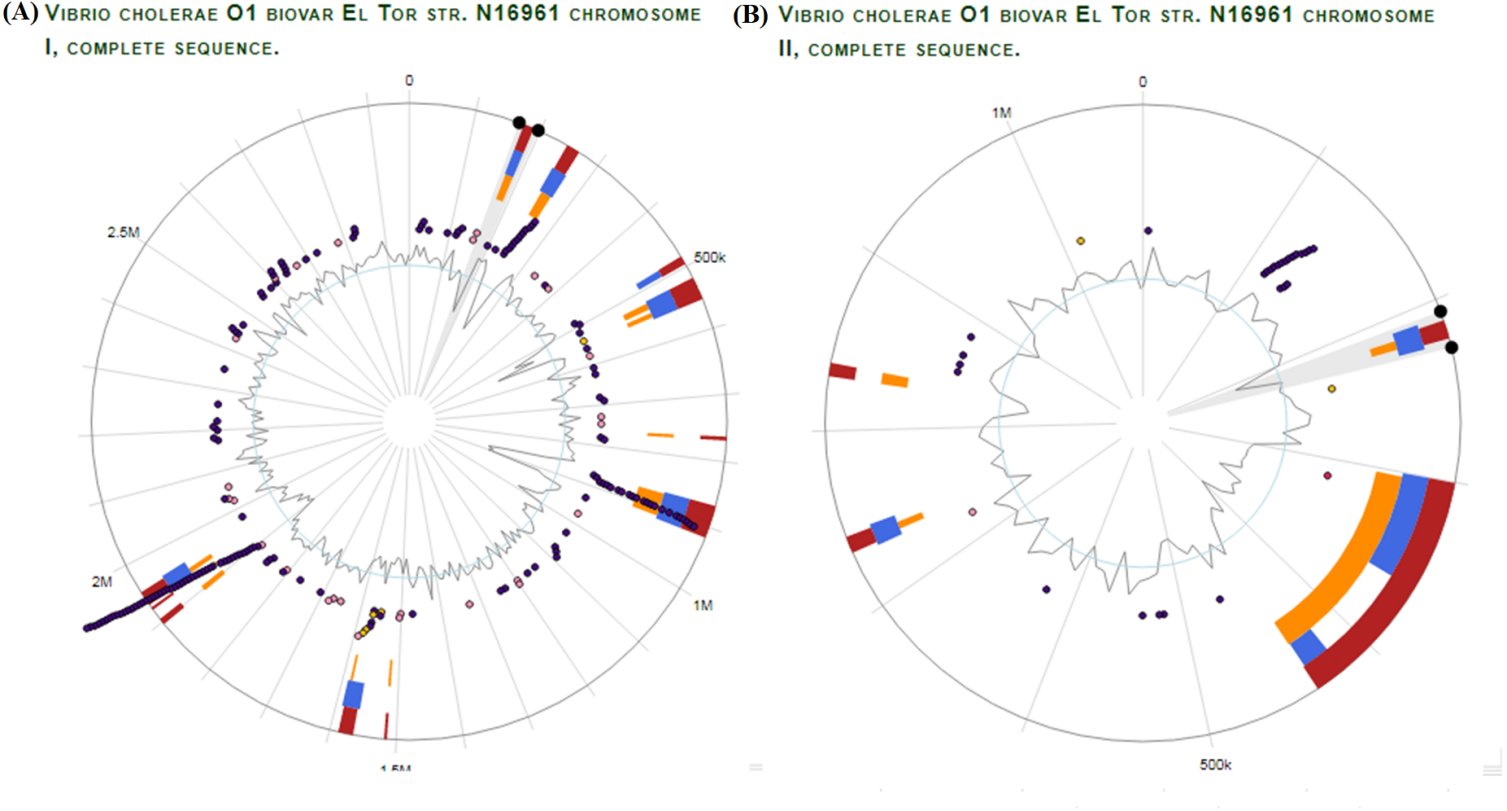
*V. Cholerae* O1 El Tor Genomic Analysis for virulence and antibiotic resistance genes. Dark and light purple dots represent the curated virulence factors and antibiotic resistance genes respectively, in *V. cholerae* chromosome I (A) and chromosome II (B). The orange and blue bars represent the algorithms used by server for prediction of genomic islands i.e., SIGI-HMM, IslandPath-DIMOB respectively. The red bar represents the integrated results for all the methods used. The inner most circle indicates the GC skew for both chromosomes. The figure was generated using IslandViewer 3 (http://www.pathogenomics.sfu.ca/islandviewer/).

We selected *V. Cholerae* O1 biovar El tor str. N16961 as a reference strain for our vaccine prediction strategy. Unlike other prokaryotes, *V. cholerae* contains two circular chromosomes. It is a unique biotype due to hemolysin production. Using the virulence factor database (VFDB), proteomic data of virulent strain of *V. Cholerae* was obtained. Genomic visualization of curated virulence factors was performed using the server ‘*Island Viewer 4: An integrated interface for computational identification and visualization of genomic islands* (http://www.pathogenomics.sfu.ca/islandviewer/), as shown in Fig. 2 (Bertelli et al., 2017).

### Subcellular localization of screened targets

**Figure 3:**
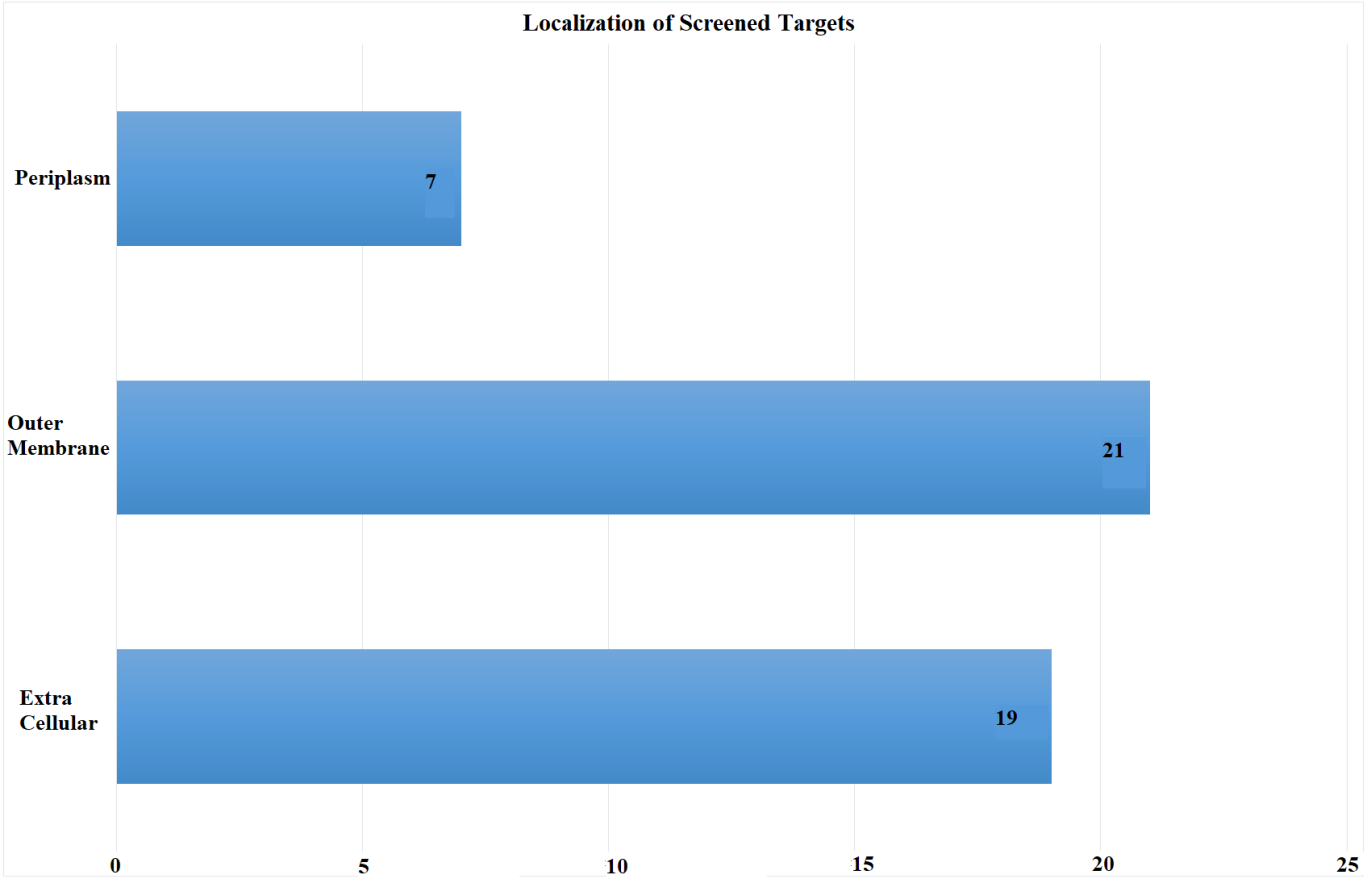
Subcellular Localization of Screened Targets. Screening process yielded total of 47 proteins as potential vaccine candidates. Subcellular localization yielded after CELLO analysis (EC, extracellular; OM, outer membrane; P, periplasmic).

Subcellular localization is the most critical screening criterion. Antigens exposed at the surface are more accessible to immune system. We scrutinized proteins exposed at pathogen’s surface with potential role as antibiotic resistance determinants. Proteomic sequences were subjected to subcellular localization analysis which is a crucial step in screening out potent vaccine candidates’ identification. The proteome was screened based on subcellular location, number of transmembrane helices and minimum adhesion probability. In total, 47 proteins (Table S1) were predicted as potential vaccine candidates consisting of 21 outer membrane, 19 extracellular and seven periplasmic proteins as shown in Fig. 3. These proteins had less than 1 transmembrane helices and an adhesion probability greater than 0.51, the cut off value to assign a protein as an adhesin. Moreover, these proteins showed no similarity to human proteins (Sachdeva et al., 2004).

The antigenicity scores were predicted using the VaxiJen v2.0 server to further refine the selection. This software predicted antigenicity of proteins from FASTA-submitted amino acid sequences based on their physiochemical properties. This feature is characterized according to an antigenic score. Our Vaxijen analysis predicted 45 antigenic potential vaccine candidates out of 47 proteins with antigenicity scores greater than 0.41. An antigenicity score of over 0.40 indicates protein antigenicity (Doytchinova & Flower, 2007). To be more specific, we selected proteins giving the antigenicity score equal to or greater than 0.7. As a result we obtained five prioritized proteins (details in Table 1).

**Table 1:** Details of predicted *V. cholerae* vaccine candidates based on genome sequence analysis.

**Protein accession**	**Protein name**	**Localization**	**Adhesin Probability**	**Trans- membrane helices**	**Antigenicity**	**Pfam domains**	**Functional discription**
NP_230184.1	Lipoprotein NlpD	Outer membrane	0.654	0	0.7878	PF01476	Membrane protein
NP_230282.1	Outer membrane protein OmpU	Outer membrane	0.563	0	0.74	PF00267	Outer membrane protein (porin)
NP_230492.1	Accessory colonization factor AcfA	Outer membrane	0.550	0	0.7709	PF13505	ATPase-coupled sulfate transmembrane transporter activity
NP_231488.1	Porin, putative	Outer membrane	0.518	0	0.7463	PF13609	Outer membrane protein (porin
NP_233253.1	Outer membrane protein W	Outer membrane	0.640	0	0.7774	PF03922	Outer membrane protein

### PPI interactions and COG analysis

The predicted proteins were studied for their potential biological roles and proteomic interactions. STRING (Search Tool for the Retrieval of Interacting Genes/Proteins) provides essential information regarding interactions of desired proteins (Szklarczyk et al., 2010).

**Figure 4:**
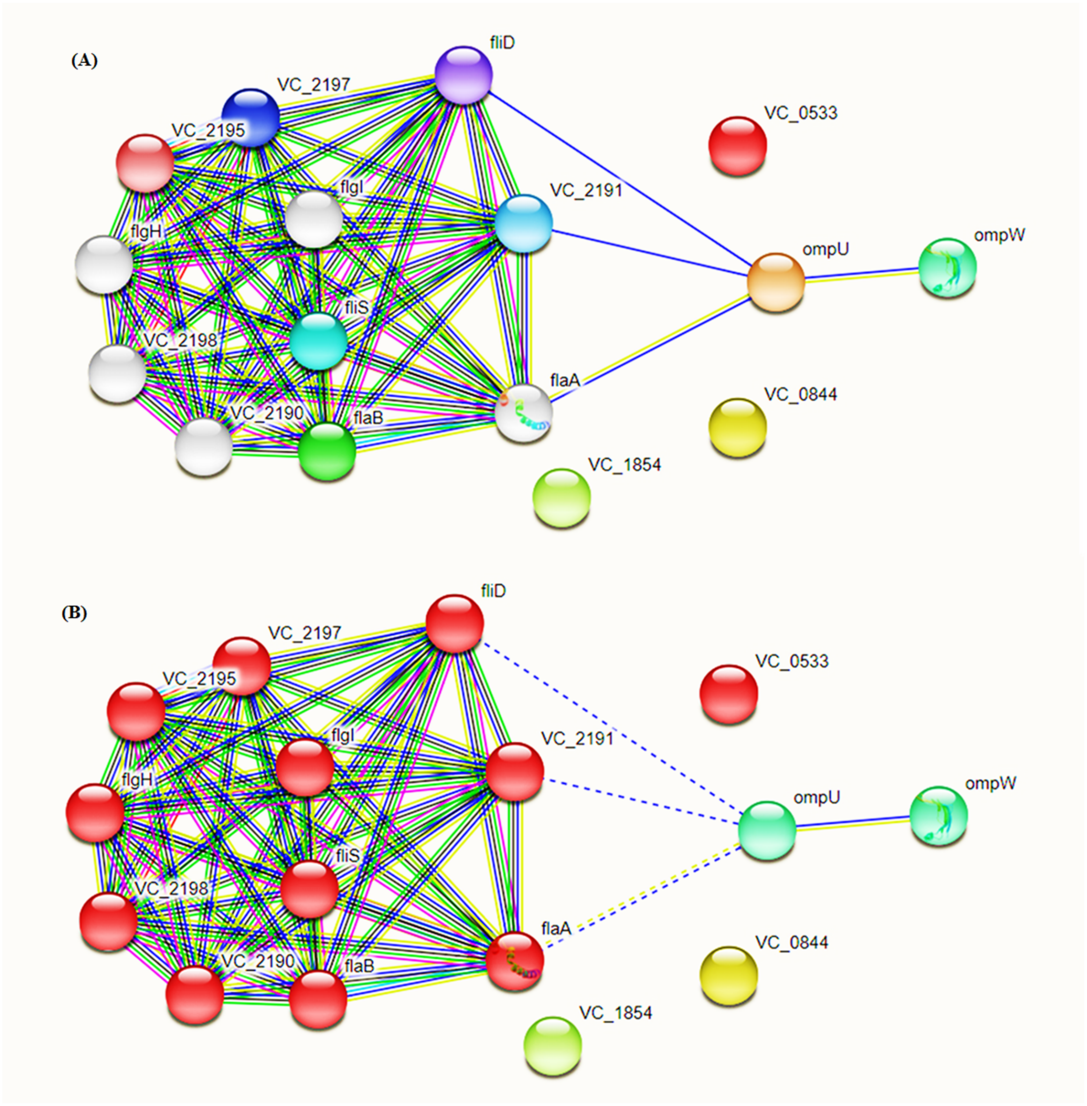
Protein–Protein Interaction Analysis. Prioritized protein targets were subjected to PPI assessment using the STRING database. Interaction suggests involvement of OmpC and OmpW in flagellar development (A) and adhesion (B).

Intra-specie protein-protein interactions were calculated for the selected proteins using STRING database online network analysis tools (Fig. 4). The STRING database also provided the Cluster of Orthologous Groups (COG) analysis tools. COGs analysis was conducted on the basis of protein sequence similarity and conserved domains in comparison to reported proteins in the database.

For COG protein functional categorization, four out of the given five proteins fall into “outer membrane/membrane” group whereas 1 was identified as ATPase-coupled sulfate with transmembrane transporter activity.

### 3D structures of prioritized vaccine candidates

**Figure 5:**
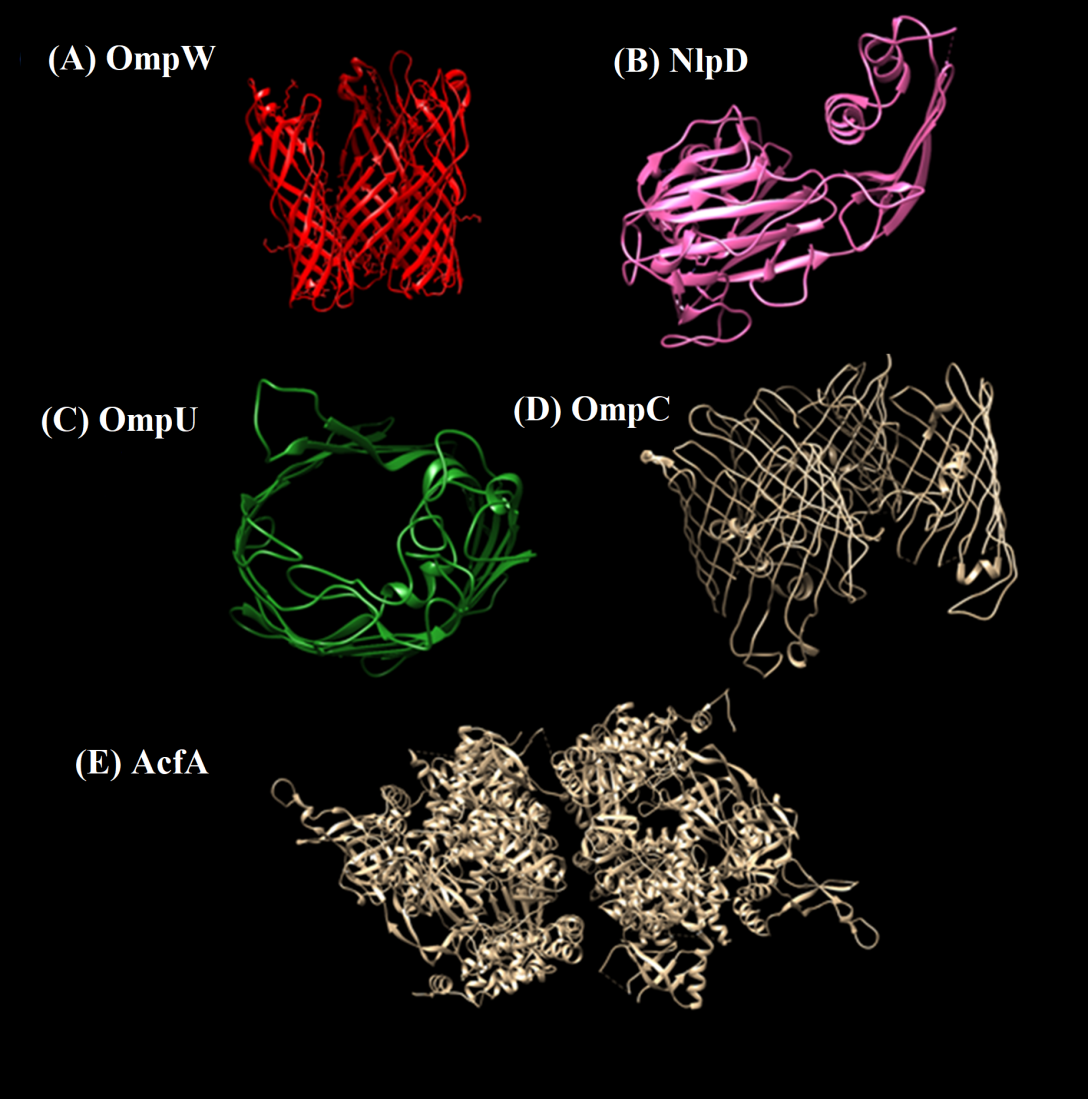
3D structures of prioritized proteins. (A) OmpW, (B) NlpD, (C) OmpU, (D) OmpC, (E) AcfA. Predicted and crystal structures of prioritized proteins. NlpD had predetermined crystal structure in PDB which was retrieved (PDB id 2gu1). For the rest of the proteins, homology models were predicted using the Swiss Model server.

Structural information is vital for proteinaceous targets before predicting immunogenic domains. Availability of crystalline structures for the selected protein was checked in experimental structural database Protein Data Bank (PDB) (Bernstein et al., 1978). One crystalline structure available for protein NlpD (PDB id 2gu1) was retrieved. For other selected proteins suitable templates were searched within PDB. Protein structures were predicted using SwissModel server via homology modelling approach. 3D structures of the Prioritized protein targets are given in Fig. 5.

### Predicted prioritized vaccine targets

#### Epitope mapping

Peptide vaccines are more convenient and safer than the contemporary vaccines. As it includes only the immunogenic epitopes rather than full three dimensional structures obtained from pathogens. Immunogenic potential is primarily dependent on Major Histocompatibility Complex (MHC) binding affinity. Thus predicting the epitopes with higher binding potential for MHCs is necessary to design peptide vaccines (Naz et al., 2015). The prioritized proteins were subjected to primary sequence based antigenic and virulence epitopes prediction. Since there are multiple algorithms for prediction of antigenic epitopes, thus multiple servers were used for evaluation of selected vaccine candidates. Primary sequences of the proteins were subjected to alignment independent antigenic prediction based on physicochemical properties of proteins. Proteins having score >0.4 were considered antigenic. The resultant antigenic proteins were subjected to further studies.

Out of the 47 predicted *V. cholerae* vaccine candidates, proteins with the antigenicity score greater than 0.7 were filtered through VaxiJen 2.0. Only the epitopes with *P* value greater than 0.9 were selected for each protein and antigenicity scores were further analyzed specific for all epitope sequences. MHCPred was used for antigenicity and IC_50_ calculation for the selected epitopes  (Guan et al., 2003). MHCPred covers a range of different human MHC allele peptide specificity models. These include Class I (HLA-A*0101, HLA-A*0201, HLA-A*0202, HLAA*0203, HLA-A*0206, HLA-A*0301, HLA-A*1101, HLAA*3301, HLA-A*6801, HLA-A*6802 and HLA-B*3501) and Class II (HLA-DRB1*0101, HLA-DRB1*0401 and HLADRB1* 0701) alleles (Guan et al., 2003). Moreover, MHC II epitopes were studied in detail using EpiDOCK that predicts binding to the 23 most frequent human MHC class II proteins: 12 HLA-DR, 6 HLA-DQ and 5 HLA-DP proteins. These alleles cover more than 95% of the human population. EpiDOCK is freely accessible at: http://epidock.ddg-pharmfac.net/. The epitopes were prioritized based on the number of binding alleles to the given epitope sequences. Consequently, 10 epitopes were prioritized (Table 2).

**Table 2:** Prioritized *V. cholerae* vaccine candidates based on epitope mapping.

**#**	**MHC class**	**Index**	**Epitope**	**Antigenicity**
1	MHCI	6	LYSFRLGLLL	1.4424
	MHCII	3	GLLLFCSLL	1.5179
2	MHCI	3	YSDNGEDGY	1.6821
	MHCI	6	SYISYQFNL	1.8449
	MHCII	3	YISYQFNLL	1.5362
3	MHCI	6	ALFSLGLDY	1.6604
	MHCII	6	FSFEINYSS	1.5186
4	MHCI	2	YGDGTTLGY	1.8772
	MHCI	3	RTRNSHIKK	1.9183
6	MHCII	1	TFMVQYYFG	1.4084

**Figure 6:**
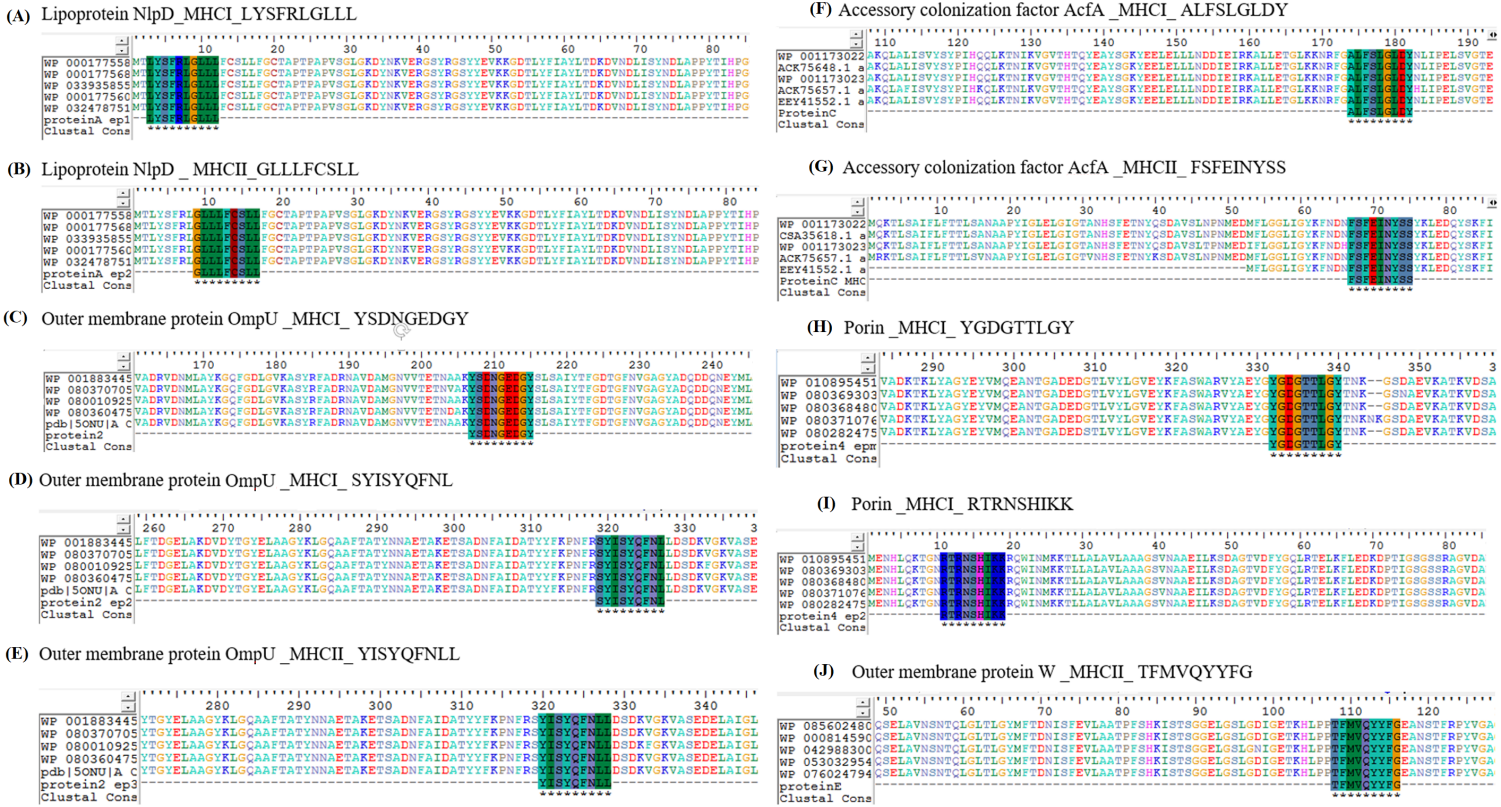
Multiple protein sequence alignment of protein targets was performed among 100 members of *V. cholerae* NCBI Taxonomic group (TAXID: 666). (A), (C), (D), (F), (H) and (I) depict the MHC-I epitopes of the selected vaccine candidate proteins while (B), (E), (G) & (J) denote MHC-II epitopes. Epitopes were found conservative in all the members of the Taxonomic group. Colors indicate the following: red, residue ED; yellow, residue G; blue, residue KR; turquoise, residue YFA; magenta, residue H; green, residue LIMV; grey, others. The sign “–”(dash) means no amino acid aligned.

The prioritized epitopes were aligned with the available strains of *V*. cholera. Sequences of the potential targets were obtained from 100 members of *V. cholerae* NCBI Taxonomic group (TAXID: 666). The predicted antigenic regions were analyzed via BioEdit Sequence Alignment Editor, for sequence divergence against *V. cholerae* representative strains and consensus sequences were obtained for respective vaccine candidate. The multiple sequence alignment for the selected epitopes showed that these peptide sequences are conserved in *V. cholerae*, as shown in Fig. 6.

## Discussion

In this study, we adopted reverse vaccinology based reductive screening and fished out five immunogenic proteins harboring 10 peptide epitopes as potential vaccine candidates in the *V. cholerae* proteome. Reverse vaccinology is a genome/proteome based approach for vaccine development that has been proved effective (Giuliani et al., 2006). Reductive screening is performed based on parameters i.e., protein essentiality, subcellular localization, host homology and effective immunogenicity for predicting an effective vaccine candidate. A computer-aided screening process is more convenient, accurate and fast in comparison with the contemporary vaccine development which depends on a hit and trial approach. This strategy studies key aspects of the pathogen i.e., genome, essential metabolism, virulence and protein-protein interactions and incorporates this information for determining the prospective vaccine candidates prior to any wet lab experimentations (Naz et al., 2015). One of the key limitation is that this strategy is primarily focused on prediction of peptide epitopes based on amino acid sequences of the proteins. Hence, the long known immunogenic potential of nonprotein antigens (i.e., Lipopolysaccharides) couldn’t be accounted in this strategy  (Lüderitz et al., 1982; McGhee et al., 1980; Del Barrio et al., 2015). But the addition of such known epitopes as adjuvants is a good approach for overcoming this limitation (Caucheteux et al., 2017; Noguchi et al., 2017). Another prominent limitation could be the high mutation rate of the viral surface proteins (Steinhauer & Holland, 1987; Echave, Spielman & Wilke, 2016). The prospects of reverse vaccinology approach have been discussed in detail in our previous study (Rashid et al., 2017).

Peptide vaccines theoretically have several advantages over conventional and recently developed DNA vaccines (Ingolotti et al., 2010). Lesser cost and convenient synthesis with improved safety and stability are the key features which have been demonstrated in various studies (Firbas et al., 2006; Jagannath et al., 2009). Conventional vaccines are overburdened with unnecessary antigens which divert immune response resources thus might result in a chaos which lacks the required dedicated for eliminating the threat thus impedes the vaccine efficacy (Czerkinsky & Holmgren, 2015). As in case of cholera, whole cell vaccines were only able to impart varying protective efficiency (39–60%) in studies conducted in Bangladesh and Vietnam (Clemens et al., 1990; Thiem et al., 2006). While live attenuated vaccine was unsuccessful in generating long term protective response (Fournier, 1998). One interesting inconsistency is the comparative efficacy of cholera vaccines in developed and developing countries  (Czerkinsky & Holmgren, 2009), while a notable recent exception was observed in South Sudan  (Bekolo et al., 2016). Considering these factors, the need for novel strategy is vital to achieve protection against this pathogen.

Reported prioritized targets included lipoprotein *NlpD*, outer membrane protein *OmpU*, accessory colonization factor *AcfA*, putative porin, and outer membrane protein *OmpW*. These predicted proteins are involved in important virulence mechanisms of *V. cholerae*. Role of lipoprotein *NlpD*, has been studied in reference to cell division and intestinal colonization by the pathogen. Septal peptidoglycan (PG) amidase, *AmiB* is involved in separation of daughter cells at the end of cell division process (Yakhnina, McManus & Bernhardt, 2015). *AmiB* is regulated by *NlpD* in *V. cholerae* (Möll et al., 2014). Both of these processes are important for pathogen’s survival in host intestine. Another predicted potential target accessory colonization factor *AcfA* is of peculiar interest as it has been subjected to edible vaccine (Sharma et al., 2008). Targeting *NlpD* and *AcfA* could provide passive therapeutic potential as immune inactivation would impede the pathogen’s ability to colonize and multiply in the small intestine.

Among these vaccine candidates, we obtained two outer membrane proteins (OMPs), *OmpU and OmpW* that also serve as antibiotic resistance determinants. In vibrio species OMPs are studied to play vital roles as porins in iron, phosphate and sugar acquisition as well as in bacterial attachment to solid surfaces (Aeckersberg et al., 2001). While *OmpU* has been reported to be involved in conferring polymyxin B sulfate resistance  (Mathur & Waldor, 2004). We consider *OmpU* as an important vaccine candidate selected via our computational framework as it is not only involved in host cell invasion but also confers antibiotic resistance (Duperthuy et al., 2011). Moreover, it has also been used as an effective vaccine candidate in other vibrio species such as *V. alginolyticus* and *V. harveyi* in *Lutjanus erythropterus* and *Scophthalmus maximus*, respectively (Cai et al., 2013; Wang et al., 2011). Such studies provide good examples of how a reverse vaccinology strategy can be used for systematic vaccine design against drug resistant microbial pathogens.

Another important predicted potential vaccine candidate is *OmpW*. It’s a characteristic outer membrane protein expressed by *V. cholerae* and has been used to identify infectious agent via different PCR based detection techniques. Studies have reported this protein to be conserved and harbors immunogenic properties (Nandi et al., 2005; Jalajakumari & Manning, 1990). Considering its abilities, *OmpW* could be a good candidate for developing a broad spectrum and effective vaccine.

Interestingly, when we analyzed our screened results with a recent antibody profiling study of the *V. cholerae* O1 protein immunome, nine overlapping antigens were observed (Charles et al., 2017). These antigens were: Organic solvent tolerance protein (VC0446), outer membrane protein *OmpU* (VC0633), toxin co-regulated pilin (VC0828), outer membrane protein *OmpV* (VC1318), neuraminidase (VC1784), hemolysin-related protein (VC1888), and flagellar proteins/components (VC2142, VC2143, VC2187). Among these nine, outer membrane protein OmpU (VC0633) was common in the most effective antigens reported in the final selection of the both studies. While toxin co-regulated pilin (VC0828) was among our initial screening list but it is reported as one of the most effective by Charles et al. (2017). One possible reason of the screening results could be the difference in the adopted screening strategies. Our strategy was purely computational, with the calculations all derived using only the peptide sequences of the proteins. A shortcoming to this is that it could only be applied for peptide antigens, while on the other hand antigens other than proteins do have their immuno-protective potential i.e., the O polysaccharide, LPS, etc. These overlapping proteins in the two investigations provide confidence to our prediction.

## Conclusion

With the aim of finding new protective antigens for vaccine development, in this study we report on lipoprotein NlpD, outer membrane protein OmpU, accessory colonization factor AcfA, Porin, putative and outer membrane protein OmpW as potential candidates qualifying for all the set criteria. These predicted epitopes can offer a potential for the development of a reliable peptide or subunit vaccine for *V. cholerae*.

##  Supplemental Information

10.7717/peerj.6223/supp-1 Supplemental Information 1 Screening of prioritized vaccine candidates for *V. cholerae* Each sheet contains step by step prioritizatition of the selected vaccine candidates.

10.7717/peerj.6223/supp-2 Table S1 Genome: Vibrio cholerae O1 biovar El tor str. N16961

## Additional Information and Declarations

### Data Availability

The following information was supplied regarding data availability: Raw data are provided in the Supplemental Files.
