# Supplementary material for: Fishing for vaccines against Vibrio cholerae using in silico pan-proteomic reverse vaccinology approach
Source: PeerJ. 2019 Jun 19;7:e6223. doi: 10.7717/peerj.6223 (PMC6589079; doi:10.7717/peerj.6223)
Supplement: Table S1 [file peerj-07-6223-s002.docx]

| Genome: Vibrio cholerae O1 biovar eltor str. N16961 | | | |  |  |  |  |  |  |  |  |
| --- | --- | --- | --- | --- | --- | --- | --- | --- | --- | --- | --- |
| Subcellular Localization: Cellwall, Extracellular, OuterMembrane, Periplasmic | | | | | | |  |  |  |  |  |
| Maximum Number of Transmembrane Helices: 1 | | | |  |  |  |  |  |  |  |  |
| Minimum Adhesin Probability: 0.51 | | |  |  |  |  |  |  |  |  |  |
| No Similarity to Human Proteins: checked | | | |  |  |  |  |  |  |  |  |
| # | Protein Accession | Protein GI | Gene Symbol | Locus Tag | Gene ID | Protein Note | Localization | Probability | Adhesin Probability | Trans-membrane helices | Antigenacity |
| 1 | NP_229748.1 | 15640121 | VC0089 | VC0089 | 2615828 | cytochrome c551 peroxidase | Periplasmic | 0.976 | 0.566 | 0 | 0.5075 |
| 2 | NP_229813.1 | 15640186 | VC0156 | VC0156 | 2612956 | vitamin B12 receptor | Outer Membrane | 1 | 0.545 | 0 | 0.6571 |
| 3 | NP_230100.1 | 15640473 | VC0446 | VC0446 | 2615778 | organic solvent tolerance protein | Outer Membrane | 1 | 0.574 | 0 | 0.6122 |
| 4 | NP_230129.1 | 15640502 | VC0475 | VC0475 | 2615269 | enterobactin receptor protein | Outer Membrane | 1 | 0.540 | 0 | 0.6836 |
| 5 | NP_230184.1 | 15640555 | VC0533 | VC0533 | 2615202 | lipoprotein NlpD | Outer Membrane | 0.992 | 0.654 | 0 | 0.7878 |
| 6 | NP_230282.1 | 15640653 | VC0633 | VC0633 | 2615421 | outer membrane protein OmpU | Outer Membrane | 1 | 0.563 | 0 | 0.74 |
| 7 | NP_230476.1 | 15640845 | VC0828 | VC0828 | 2614495 | toxin co-regulated pilin | Extracellular | 1 | 0.607 | 1 | 0.3 |
| 8 | NP_230492.1 | 15640861 | VC0844 | VC0844 | 2614511 | accessory colonization factor AcfA | Outer Membrane | 0.952 | 0.550 | 0 | 0.7709 |
| 9 | NP_230576.1 | 15640945 | VC0929 | VC0929 | 2614149 | hypothetical protein VC0929 | Extracellular | 0.964 | 0.620 | 0 | 0.4824 |
| 10 | NP_230577.1 | 15640946 | VC0930 | VC0930 | 2614150 | hemolysin-related protein | Extracellular | 0.844 | 0.551 | 0 | 0.6024 |
| 11 | NP_230582.1 | 15640951 | VC0935 | VC0935 | 2614155 | hypothetical protein VC0935 | Outer Membrane | 0.949 | 0.628 | 0 | 0.6315 |
| 12 | NP_230619.1 | 15640988 | VC0972 | VC0972 | 2614225 | porin, putative | Outer Membrane | 0.952 | 0.511 | 0 | 0.6753 |
| 13 | NP_230687.1 | 15641055 | VC1042 | VC1042 | 2614312 | long-chain fatty acid transport protein | Outer Membrane | 0.993 | 0.692 | 1 | 0.5406 |
| 14 | NP_230688.1 | 15641056 | VC1043 | VC1043 | 2614313 | long-chain fatty acid transport protein | Outer Membrane | 1 | 0.682 | 0 | 0.5614 |
| 15 | NP_230720.1 | 15641088 | VC1075 | VC1075 | 2614345 | hypothetical protein VC1075 | Periplasmic | 1 | 0.653 | 0 | 0.5635 |
| 16 | NP_230852.1 | 15641220 | VC1207 | VC1207 | 2614640 | hypothetical protein VC1207 | Outer Membrane | 0.949 | 0.522 | 0 | 0.5913 |
| 17 | NP_230962.1 | 15641330 | VC1318 | VC1318 | 2614772 | outer membrane protein OmpV | Outer Membrane | 1 | 0.682 | 0 | 0.4878 |
| 18 | NP_230973.1 | 15641341 | VC1329 | VC1329 | 2614783 | opacity protein-related protein | Outer Membrane | 0.952 | 0.541 | 0 | 0.4856 |
| 19 | NP_231006.1 | 15641374 | VC1362 | VC1362 | 2614816 | amino acid ABC transporter, periplasmic amino acid-binding protein | Periplasmic | 0.976 | 0.581 | 0 | 0.5473 |
| 20 | NP_231058.1 | 15641426 | VC1415 | VC1415 | 2614047 | hcp protein | Extracellular | 0.971 | 0.595 | 0 | 0.6465 |
| 21 | NP_231419.1 | 15641787 | VC1784 | VC1784 | 2613664 | neuraminidase | Extracellular | 1 | 0.531 | 0 | 0.6254 |
| 22 | NP_231449.1 | 15641817 | VC1815 | VC1815 | 2613695 | C factor cell-cell signaling protein | Extracellular | 0.971 | 0.649 | 0 | 0.4858 |
| 23 | NP_231488.1 | 15641856 | VC1854 | VC1854 | 2613608 | porin, putative | Outer Membrane | 1 | 0.518 | 0 | 0.7463 |
| 24 | NP_231522.1 | 15641890 | VC1888 | VC1888 | 2613517 | hemolysin-related protein | Extracellular | 1 | 0.542 | 0 | 0.5126 |
| 25 | NP_231586.1 | 15641954 | VC1952 | VC1952 | 2613456 | chitinase | Extracellular | 1 | 0.672 | 0 | 0.5629 |
| 26 | NP_231773.1 | 15642141 | VC2142 | VC2142 | 2613278 | flagellin | Extracellular | 1 | 0.524 | 0 | 0.8273 |
| 27 | NP_231774.1 | 15642142 | VC2143 | VC2143 | 2613279 | flagellin | Extracellular | 1 | 0.548 | 0 | 0.7362 |
| 28 | NP_231818.1 | 15642186 | VC2187 | VC2187 | 2613227 | flagellin | Extracellular | 1 | 0.567 | 0 | 0.7056 |
| 29 | NP_231825.1 | 15642193 | flgH | VC2194 | 2613234 | flagellar basal body L-ring protein | Outer Membrane | 0.992 | 0.520 | 0 | 0.7073 |
| 30 | NP_231828.1 | 15642196 | flgE | VC2197 | 2613237 | flagellar hook protein FlgE | Extracellular | 0.971 | 0.627 | 0 | 0.6287 |
| 31 | NP_231872.1 | 15642239 | VC2241 | VC2241 | 2613163 | cytochrome c554 | Periplasmic | 1 | 0.539 | 0 | 0.532 |
| 32 | NP_231936.1 | 15642303 | VC2305 | VC2305 | 2613101 | outer membrane protein OmpK | Outer Membrane | 1 | 0.600 | 0 | 0.6457 |
| 33 | NP_232053.1 | 15642420 | VC2423 | VC2423 | 2612965 | fimbrial protein | Extracellular | 1 | 0.633 | 1 | 0.5221 |
| 34 | NP_232418.1 | 15600788 | VCA0017 | VCA0017 | 2612496 | hcp protein | Extracellular | 0.971 | 0.595 | 0 | 0.6465 |
| 35 | NP_232595.1 | 15600965 | VCA0195 | VCA0195 | 2612373 | hypothetical protein VCA0195 | Outer Membrane | 0.952 | 0.736 | 0 | 0.6485 |
| 36 | NP_232601.1 | 15600971 | VCA0201 | VCA0201 | 2612359 | hypothetical protein VCA0201 | Extracellular | 0.964 | 0.605 | 0 | 0.4455 |
| 37 | NP_232617.1 | 15600987 | VCA0218 | VCA0218 | 2612876 | thermolabile hemolysin | Extracellular | 1 | 0.597 | 0 | 0.4107 |
| 38 | NP_232626.1 | 15600996 | VCA0227 | VCA0227 | 2612388 | iron(III) ABC transporter, periplasmic iron-compound-binding protein | Periplasmic | 0.976 | 0.518 | 0 | 0.5024 |
| 39 | NP_232966.1 | 15601335 | VCA0576 | VCA0576 | 2612698 | heme transport protein HutA | Outer Membrane | 1 | 0.553 | 0 | 0.6309 |
| 40 | NP_233014.1 | 15601383 | VCA0625 | VCA0625 | 2612781 | TonB receptor-related protein | Outer Membrane | 1 | 0.541 | 0 | 0.5823 |
| 41 | NP_233036.1 | 15601405 | VCA0647 | VCA0647 | 2612266 | hypothetical protein VCA0647 | Periplasmic | 0.984 | 0.655 | 0 | 0.3 |
| 42 | NP_233197.1 | 15601566 | VCA0811 | VCA0811 | 2611865 | N-acetylglucosamine-binding protein A | Extracellular | 1 | 0.514 | 0 | 0.7353 |
| 43 | NP_233235.1 | 15601604 | VCA0849 | VCA0849 | 2612210 | hypothetical protein VCA0849 | Extracellular | 1 | 0.665 | 0 | 0.7263 |
| 44 | NP_233248.1 | 15601617 | VCA0862 | VCA0862 | 2612849 | long-chain fatty acid transport protein | Outer Membrane | 1 | 0.710 | 0 | 0.5955 |
| 45 | NP_233251.1 | 15601620 | VCA0865 | VCA0865 | 2612874 | hemagglutinin/protease | Extracellular | 1 | 0.695 | 1 | 0.6694 |
| 46 | NP_233253.1 | 15601622 | VCA0867 | VCA0867 | 2612873 | outer membrane protein W | Outer Membrane | 1 | 0.640 | 0 | 0.7774 |
| 47 | NP_233411.1 | 15601780 | VCA1027 | VCA1027 | 2612419 | maltose operon periplasmic protein, putative | Periplasmic | 0.976 | 0.562 | 0 | 0.4966 |
